# The effects of training intervention on the prevention of knee joint injuries: a systematic review and meta-analysis

**DOI:** 10.3389/fphys.2025.1455055

**Published:** 2025-02-24

**Authors:** Gangbin Zheng, Sai Zeng, Tiangeng Li, Liang Guo, Ling Li

**Affiliations:** ^1^ School of Physical Education and Sports Science, South China Normal University, Guangzhou, China; ^2^ Department of Comprehensive Physical Education, Chencun Town Central Primary School, Foshan, China; ^3^ Department of Health and Human Performance, College of Idaho, Caldwell, United States

**Keywords:** sports intervention programs, meta-analysis, knee injury prevention, athletes, systematic review

## Abstract

**Objective:**

To evaluate the impact of neuromuscular, core strength, balance, and proprioceptive training on preventing knee injuries in young athletes, to identify optimal intervention characteristics.

**Methods:**

This review followed the 2020 guidelines of the Preferred Reporting Items for Systematic Reviews and Meta-Analyses (PRISMA). A systematic search of English and Chinese literature in databases included PubMed, Web of Science, EBSCO, CNKI, and Wanfang, covering studies published from January 1, 2000, to 12 April 2024. Inclusion criteria targeted randomized controlled trials (RCTs) on training interventions aimed at knee injury prevention among young athletes. The analysis used a random-effects model to pool data from studies meeting our criteria, ensuring a comprehensive evaluation of intervention effectiveness.

**Results:**

A total of 19 randomized controlled trials (RCTs) involving 28,176 subjects were included. The meta-analysis showed that training intervention programs reduced the risk of lower extremity knee injuries by 25% (RR = 0.75, 95% CI: 0.65–0.85). The most notable effects were observed in intervention with exercise duration of 5–15 min, frequencies of 4–5 times per week, and program lengths exceeding 26 weeks.

**Conclusion:**

The findings highlight the effectiveness of specific training interventions in reducing knee injury risk among athletes. These insights provide a clear framework for designing training routines that effectively prevent knee injuries.

## Introduction

Lower extremity injuries are highly prevalent among young athletes, with female athletes often facing a 2–8 times higher non-contact anterior cruciate ligament (ACL) injury rate compared to male athletes (Wild et al., 2012). Exercise-based interventions represent a comprehensive training approach that includes neuromuscular training, comprehensive warm-up exercises, core strength training, and specific balance training. These interventions aim to improve qualities such as strength, speed, agility, proprioception, and balance through targeted training. Currently, exercise-based preventive interventions have been widely applied in competitive sports, rehabilitation, and integrated into daily training as part of injury prevention programs (Johnson et al., 2020).

Multiple studies have demonstrated that exercise-based preventive interventions can reduce the incidence of lower extremity injuries and enhance athletic performance (Akerlund et al., 2020; Halvorsen et al., 2023; Pollard et al., 2017). However, some scholars argue that exercise-based preventive programs merely serve as pre-training warm-up routines and do not effectively prevent lower extremity injuries (Hilska et al., 2021). Consequently, there remains a certain degree of controversy regarding the effectiveness of exercise-based interventions. There remains a critical gap in understanding how the specific characteristics of these programs—duration, frequency, and intensity—affect their overall effectiveness. This gap signifies the need for a systematic evaluation that not only quantifies the impact of these variables on injury prevention but also identifies optimal program configurations for athletes across different sports. Furthermore, a consensus on the effectiveness of these interventions has not been reached.

The purpose of this meta-analysis is to identify Level 1 evidence studies on knee injury preventive interventions for athletes, evaluate the internal validity of these studies, and assess the quantitative effectiveness of these preventive programs. These interventions typically include a combination of neuromuscular, core strength, balance tailored specifically for athletes across diverse sports disciplines. The evaluation focuses on the duration, frequency, and intensity of these programs, aiming to quantitatively assess their effectiveness in reducing knee injury rates among athletes.

## Materials and methods

### Search strategy

This review followed the 2020 guidelines of the Preferred Reporting Items for Systematic Reviews and Meta-Analyses (PRISMA-ScR) checklist (Page et al., 2021) and was managed using Covidence online software. Electronic literature was systematically searched in English databases (PubMed, Web of Science, and EBSCO) and Chinese databases (CNKI and Wanfang Database) from January 1, 2000, to 12 April 2024, using key terms and their synonyms such as “Physical Activity, Sport, Training, Exercises, Acute Exercises, Aerobic Exercise, Isometric Exercise, Physical Exercise, prevent, reduces, Lower extremity injuries, Knee Injuries, Injury, Menisci, Medial Ligament of Knee, Anterior Cruciate Ligaments, Lateral collateral ligament, Cartilage, Patella, Kneecap” with the last search conducted on 12 April 2024, and all retrieved literature imported into Zotero for initial screening and further inclusion based on the PRISMA statement. The complete search strategy is provided in Supplementary Appendix A. The PICOS (patient, intervention, comparison, outcome, and study design) strategy and PRISMA checklist are provided in Supplementary Appendix A. Moreover, reference lists of included articles were also reviewed to ensure that all relevant studies meeting the selection criteria were identified.

### Selection criteria

#### Inclusion criteria

①Study type: Randomized controlled trials (RCTs). ②Participants: Young athletes with sports experience. ③Information required: Sample size, gender, age of participants. ④Intervention: Additional or alternative interventions based on the original training program. ⑤Detailed experimental design and steps. ⑥Outcome measure: Number of lower extremity sports injuries. Lower extremity injuries include anterior and posterior cruciate ligament injuries, medial and lateral collateral ligament injuries, meniscus injuries, cartilage injuries, patellar injuries, etc.

#### Exclusion criteria

①Non-randomized controlled trials, such as cohort studies. ②Review articles, animal studies, duplicate publications. ③Participants with existing diseases. ④Non-English or non-Chinese articles without full-text availability. ⑤Studies with poorly designed interventions and procedures. ⑥Studies with inconsistent outcome measures. ⑦Studies not meeting the inclusion criteria.

### Data extraction

Two researchers conducted independent and blind extraction and data entry of relevant indicators from the finally included studies.

The extracted content included author and publication year, sample size, gender, age, intervention measures, number of injuries in the experimental and control groups, total number of participants, and intervention details (exercise time, weekly frequency, exercise period).

### Quality assessment

#### Cochrane Risk of Bias assessment

The quality assessment of the included literature was performed using the Cochrane Risk of Bias assessment tool, which includes seven items: random sequence generation (A), allocation concealment (B), blinding of participants and personnel (C), blinding of outcome assessment (D), incomplete outcome data (E), selective reporting (F), and other biases (G) (Gu and Yang, 2014). Each item was scored as “yes,” “unclear,” or “no” for each article, with 1 point for “yes” and 0 points for “unclear” or “no.” Articles with a total score less than 3 were considered low-quality, 3 or 4 as moderate-quality, and 5 or more as high-quality.

#### Modified JADAD scale

The included literature was assessed for quality using the modified JADAD scale (Jadad et al., 1996)), which includes four items: randomization (generation of random sequence), allocation concealment, blinding, and description of participant withdrawal or dropout details. Each item was scored as “appropriate,” “unclear,” or “inappropriate,” with 2 points for “appropriate,” 1 point for “unclear,” and 0 points for “inappropriate.” Scores of 1-3 indicated low-quality studies, while scores of 4–7 indicated high-quality studies.

### Data analysis and processing

Review Manager (Version 5.4, Cochrane) was used for the meta-analysis. Since the incidence of lower extremity injuries is a binary variable (injured vs. non-injured) in the experimental and control groups, the risk ratio (RR) and 95% confidence interval (CI) were used as the combined effect size. An RR value not including 1 and p < 0.05 indicates a statistically significant difference between groups. An RR = 1 indicates the same risk in the experimental and control groups, RR > 1 indicates a higher risk in the experimental group, and RR < 1 indicates a lower risk in the experimental group, with values closer to 0 indicating higher protection rates (Fu et al., 2016).The I^2^ statistic reflects the proportion of heterogeneity among the total variation in effect size and is commonly used to assess the degree of heterogeneity among studies. Low, moderate, and high levels of heterogeneity are represented by I^2^ values of 25%, 50%, and 75%, respectively (Wang et al., 2009). If I^2^ ≥ 50%, there is significant heterogeneity, and a random-effects model is used. If I^2^ < 50%, a fixed-effects model is used. Additionally, the heterogeneity test also includes the Q statistic, and if P < 0.1, it indicates significant between-group differences. Sensitivity and subgroup analyses are performed to identify the sources of heterogeneity. Publication bias is assessed using a funnel plot (Tang and Liu, 2000).

## Results

### Search outcome

**FIGURE 1:**
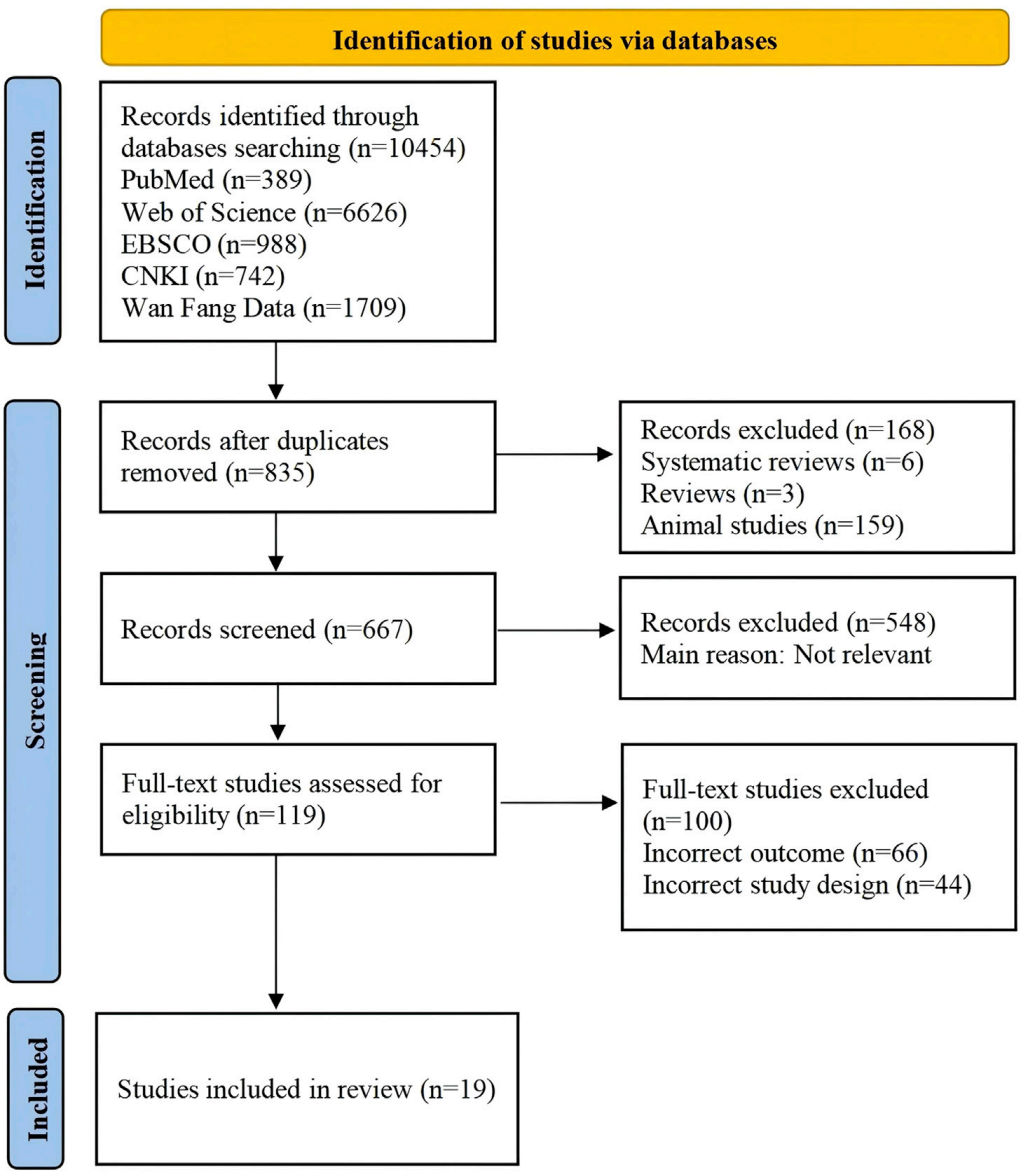
PRISMA flow chart..

Through the search of the aforementioned databases, a total of 10,454 articles were retrieved, including 8,003 in English and 2,451 in Chinese. After excluding review articles, animal studies, and duplicate publications, 667 articles remained. Following the earlier mentioned inclusion and exclusion criteria, 119 articles that clearly did met the criteria were preliminarily screened in. After carefully reading the full texts and removing articles with inconsistent outcome measures and non-randomized controlled studies, 19 articles were included in the meta-analysis. The flowchart of the literature selection process is shown in Figure 1.

### Study characteristics

A total of 19 randomized controlled trials (RCTs) were included in the meta-analysis through rigorous inclusion and exclusion criteria (see Table 1). The ages of participants ranged from 12 to 26 years, with a higher proportion of female participants. The total sample size was 28,176 individuals. The sports disciplines covered in the interventions included basketball, volleyball, soccer, handball, and other team sports. The sports interventions encompassed proprioceptive training, neural control training, strength training, balance training, core stability training, and plyometric training. The range of exercise duration was 5–30 min per session, exercise frequency ranged from 1 to 5 times per week, and the exercise period ranged from 6 to 36 weeks.

**TABLE 1:** Data extraction from included articles.

First author	Year	Total sample size EG/CG	Groups (Male/Female)	Age (years)	Intervention measures	Exercise duration (min/rep)	Exercise frequency (times/week)	Exercise period (weeks)
Aerts	2013	243 (114/129)	EG (49/41); CG (50/43)	15–26	Jump - landing training program	5–10	2	12
Emery	2007	920 (494/426)	EG (244/250); CG (220/206)	EG (13–18); CG (12–18)	Sport - specific balance training program	5–20	5	18
Gilchrist	2008	1435 (583/852)	Female	19.88	Neuromuscular and proprioceptive training programs	20–30	3	12
Toresdahl	2020	720 (352/368)	EG (107/245); CG (113/255)	EG (35.4 ± 9.1); CG (36.3 ± 9.8)	Strength training program	10	3	12
Foss	2018	474 (259/215)	Female	14 ± 1.7	Neuromuscular training program	10–25	2–3	One season
Soderman	2000	140 (62/78)	Female	EG (20.4 ± 4.6); CG (20.5 ± 5.4)	Balance board training	10–15	3	One season
Soligard	2008	1892 (1055/837)	Female	13–17	Comprehensive warm - up programme	20	2–5	32
Granelli	2017	1525 (675/850)	Male	EG (20 ± 2); CG (21 ± 1)	The FIFA 11+ injury prevention program	15–20	2–3	One season
LaBella	2011	1492 (737/755)	Female	EG (16.19 ± 1.53); CG (16.22 ± 1.06)	Neuromuscular warm - up	20	Unclear	One season
Olsen	2005	1837 (958/879)	EG (150/101); CG (808/778)	EG (16.3 ± 0.6); CG (16.2 ± 0.6)	A structured warm - up programme	15–20	1–5	32
Steffen	2013	158 (78/80)	Female	13–18	A neuromuscular injury prevention programme	20	2–3	16
Slauterbeck	2019	3611 (1825/1786)	Male and Female	15–19	The FIFA 11+ injury prevention program	15–20	2–3	One season
Bonato	2018	160 (86/74)	Female	EG (20 ± 2); CG (20 ± 1)	Neuromuscular training	30	4	32
Finch	2016	1564 (679/885)	Male	18–22	A targeted neuromuscular control exercise programme	Unclear	2	26
Steffen	2008	2020 (1073/947)	Female	13–17	The intervention program - the “11”	20	1–5	6
Walden	2012	4564 (2479/2085)	Female	EG (14.0 ± 1.2); CG (14.1 ± 1.2)	Neuromuscular training	15	2	28
Hagglund	2013	4556 (2471/2085)	Female	12–17	Neuromuscular training	15	2	24
Emery	2010	744 (380/364)	EG (219/161); CG (113/251)	13–18	A neuromuscular prevention strategy	15	3	One season
Longo	2012	121 (80/41)	Male	EG (13.5 ± 2.3); CG (15.2 ± 4.6)	The FIFA 11+ program	20	1–4	36

EG, Experimental Group; CG, Control Group.

### Evaluation of literature quality

#### Cochrane Risk of Bias assessment

**FIGURE 2:**
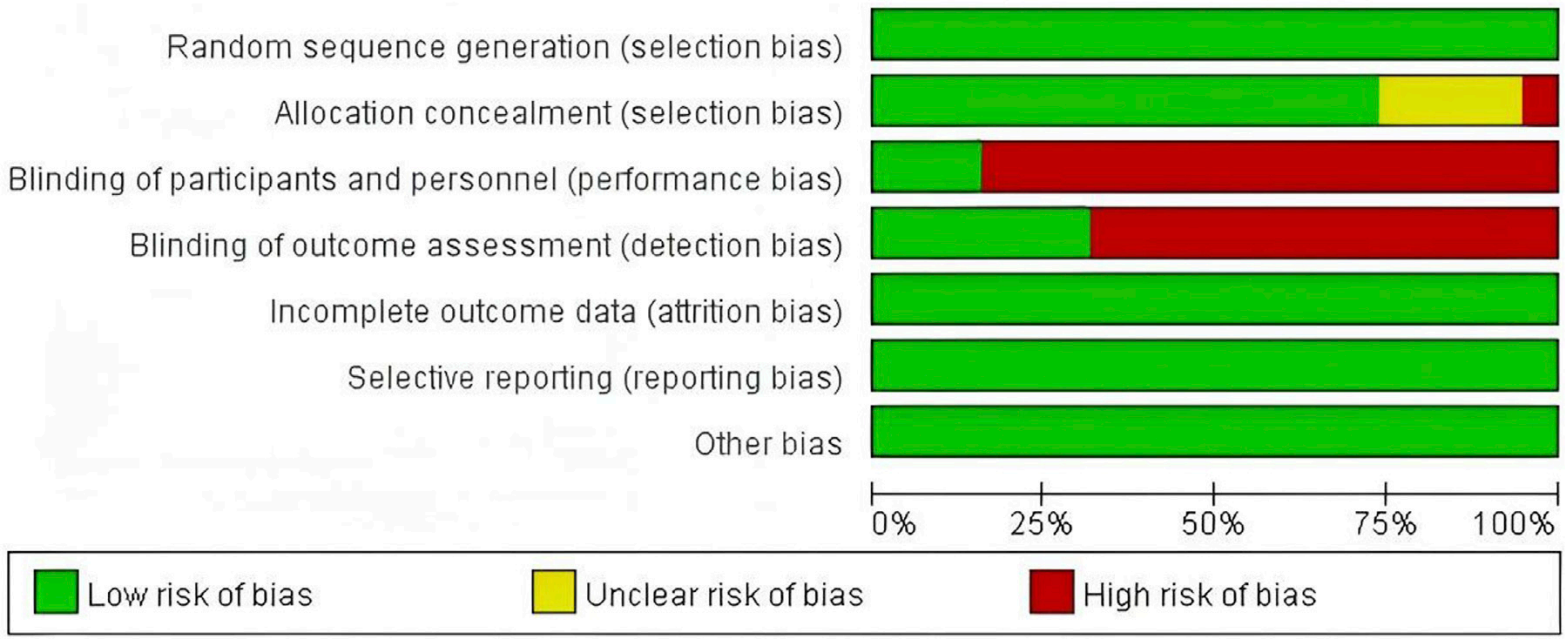
Risk of bias graph.

**FIGURE 3:**
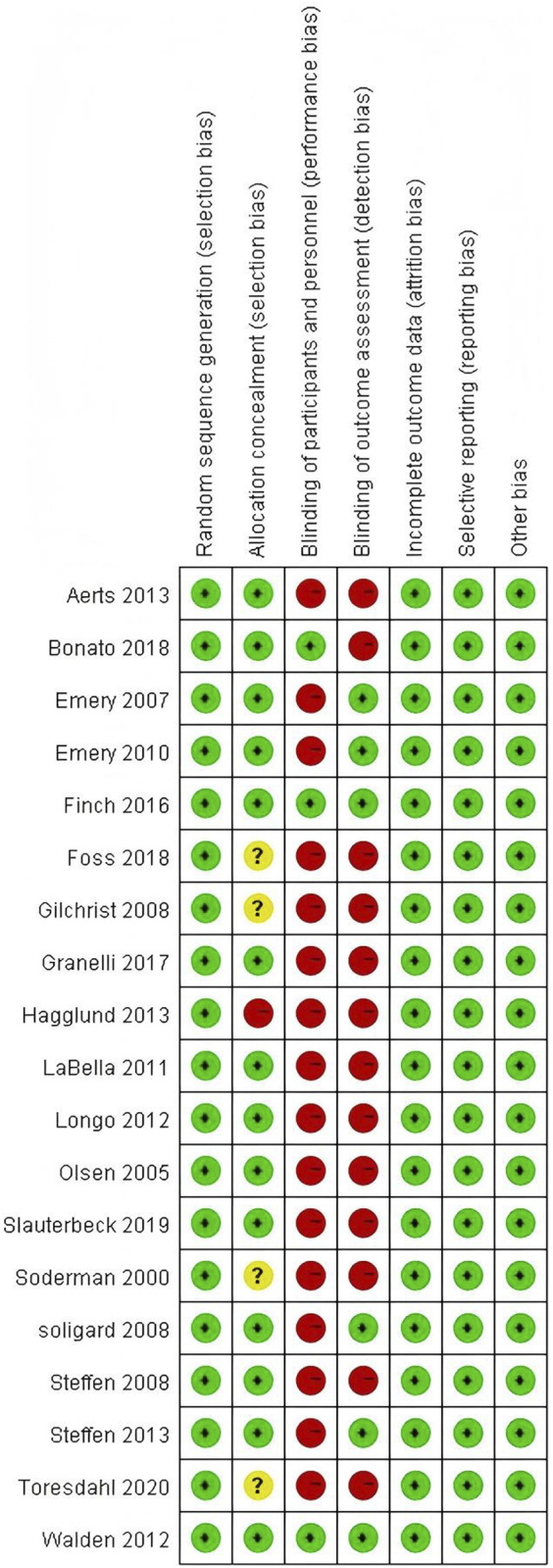
Risk of bias summary.

All 19 included articles were randomized controlled trials. All articles reported the use of random sequence generation. Nine of these detailed specific methods such as random number generation, computer-generated randomization, or block randomization, suggesting a low risk of selection bias (Aerts et al., 2013; Bonato et al., 2018; Emery et al., 2007; Emery and Meeuwisse, 2010; Finch et al., 2016; LaBella et al., 2011; Longo et al., 2012; Silvers-Granelli et al., 2017; Walden et al., 2012). However, the remaining ten articles lacked detailed descriptions of their randomization process. Allocation concealment was not described in five articles (Foss et al., 2018; Gilchrist et al., 2008; Söderman et al., 2000; Toresdahl et al., 2020). Blinding of participants and personnel was adequately reported in three articles (low risk) (Bonato et al., 2018; Finch et al., 2016; Walden et al., 2012). Blinding of outcome assessment was reported in six articles (low risk), suggesting a thorough approach to reducing detection bias (Emery et al., 2007; Emery and Meeuwisse, 2010; Finch et al., 2016; Soligard et al., 2008; Steffen et al., 2013; Walden et al., 2012). The other 13 articles provided insufficient information on blinding of assessors (unclear risk). There were no incomplete data, selective reporting, or other biases identified, indicating a low risk in these domains. Based on the assessed criteria, 14 studies were deemed to be of high quality and five of moderate quality. The bias assessment results of the included articles are presented in Figures 2, 3.

#### Modified JADAD scale score (double check results)

Nine articles reported appropriate random sequence generation, scoring 2 points, while the allocation concealment was unclear in the remaining 11 articles, scoring 1 point. Fourteen articles mentioned allocation concealment, scoring 2 points, while the remaining 6 articles had unclear allocation concealment, scoring 1 point. One article mentioned double-blinding, scoring 1 point. All 19 articles described participant withdrawal or dropout details, scoring 1 point. Among the included studies, 14 were of high quality (Aerts et al., 2013; Bonato et al., 2018; Emery et al., 2007; Emery and Meeuwisse, 2010; Finch et al., 2016; LaBella et al., 2011; Longo et al., 2012; Olsen et al., 2005; Silvers-Granelli et al., 2017; Slauterbeck et al., 2019; Soligard et al., 2008; Steffen et al., 2008; 2013; Walden et al., 2012), and 5 were of low quality (Foss et al., 2018; Gilchrist et al., 2008; Hagglund et al., 2013; Söderman et al., 2000; Toresdahl et al., 2020). Refer to Table 2 for details.

**TABLE 2:** Distribution of modified JADAD scale scores across included studies.

Literature		Random sequence production	Allocation concealment	Blinding method	Withdrawal	Total score
Aerts	2013	2	2	0	1	5
Emery	2007	2	2	0	1	5
Gilchrist	2008	1	1	0	1	3
Toresdahl	2020	1	1	0	1	3
Foss	2018	1	1	0	1	3
Soderman	2000	1	1	0	1	3
Soligard	2008	1	2	0	1	4
Granelli	2017	2	2	0	1	5
LaBella	2011	2	2	0	1	5
Olsen	2005	1	2	0	1	4
Steffen	2013	1	2	0	1	4
Slauterbeck	2019	1	2	0	1	4
Bonato	2018	2	2	0	1	5
Finch	2016	2	2	1	1	6
Steffen	2008	1	2	0	1	4
Walden	2012	2	2	0	1	5
Hagglund	2013	1	1	0	1	3
Emery	2010	2	2	0	1	5
Longo	2012	2	2	0	1	5

#### Overall effect test

**FIGURE 4:**
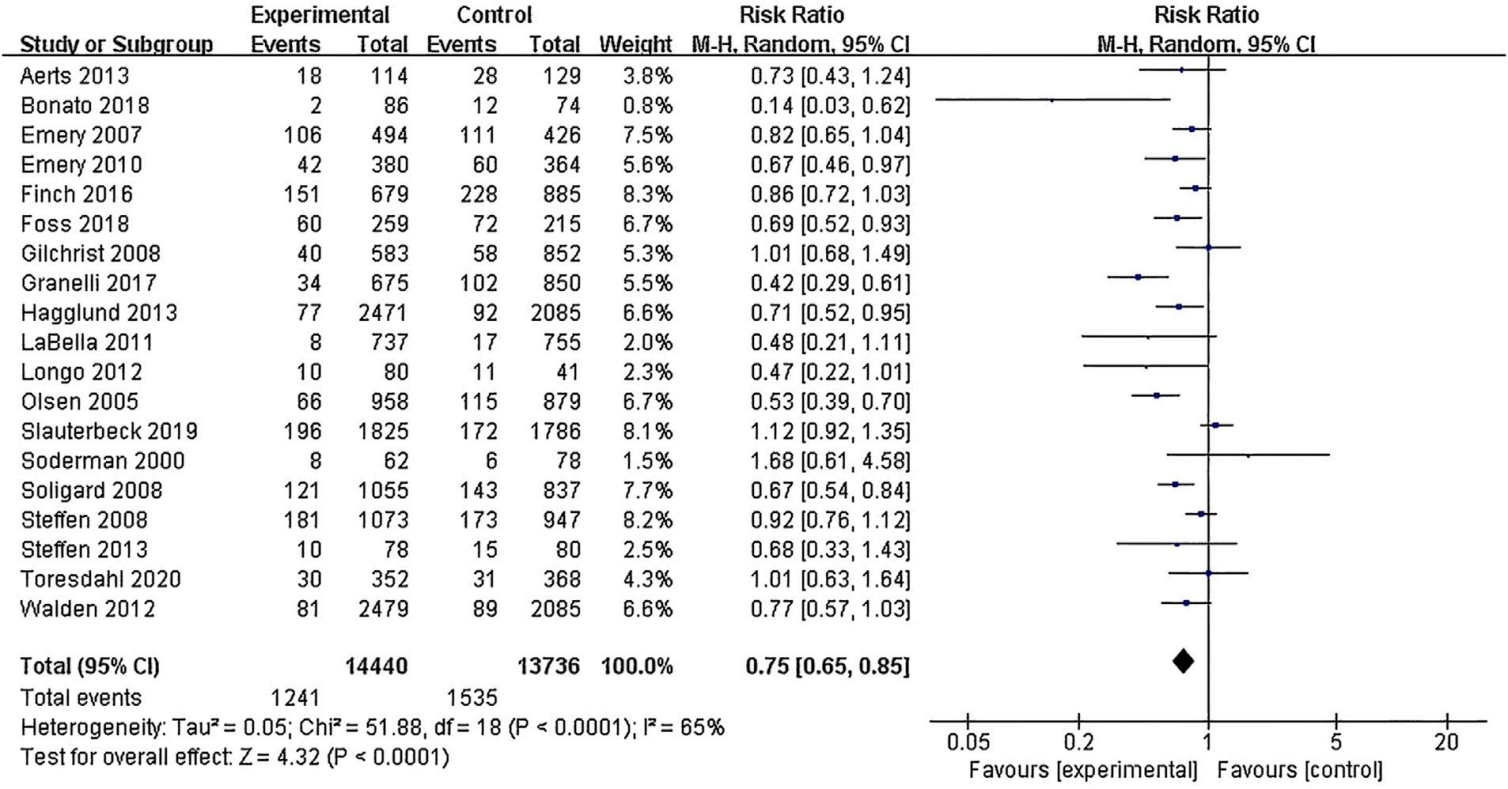
Forest plot of the overall effect test.

A total of 28,176 participants from the 19 included RCT articles were included in the overall effect test (see Figure 4). The results showed an effect size of RR = 0.75 (95% CI: 0.65–0.85; Z = 4.32; P < 0.00001), indicating that exercise interventions can effectively prevent lower limb knee injuries in young athletes. Due to I^2^ = 65% and P < 0.0001, a test for heterogeneity among the included studies was conducted, revealing moderate heterogeneity among the studies. Therefore, a random-effects model was used.

#### Sensitivity analysis

Sensitivity analysis was conducted on the 19 included articles by varying the effect size and excluding articles one by one. It was found that two articles contributed to high heterogeneity, and two articles contributed to moderate heterogeneity. By comparing them with other studies, it was discovered that one article had excessively long exercise duration of 30 min, while the other two articles combined injury results from stratified experimental groups. One of these articles involved interventions on handball athletes, indicating that the experimental design applied to the participants was the source of moderate heterogeneity among the studies. After removing these articles, the heterogeneity among the studies decreased to I^2^ = 12%. Comparing the effect sizes with and without the excluded article, the RR values remained within the original confidence interval, indicating a higher level of credibility for the meta-analysis results. Thus, these four studies can be retained.

#### Subgroup analysis results

**FIGURE 5:**
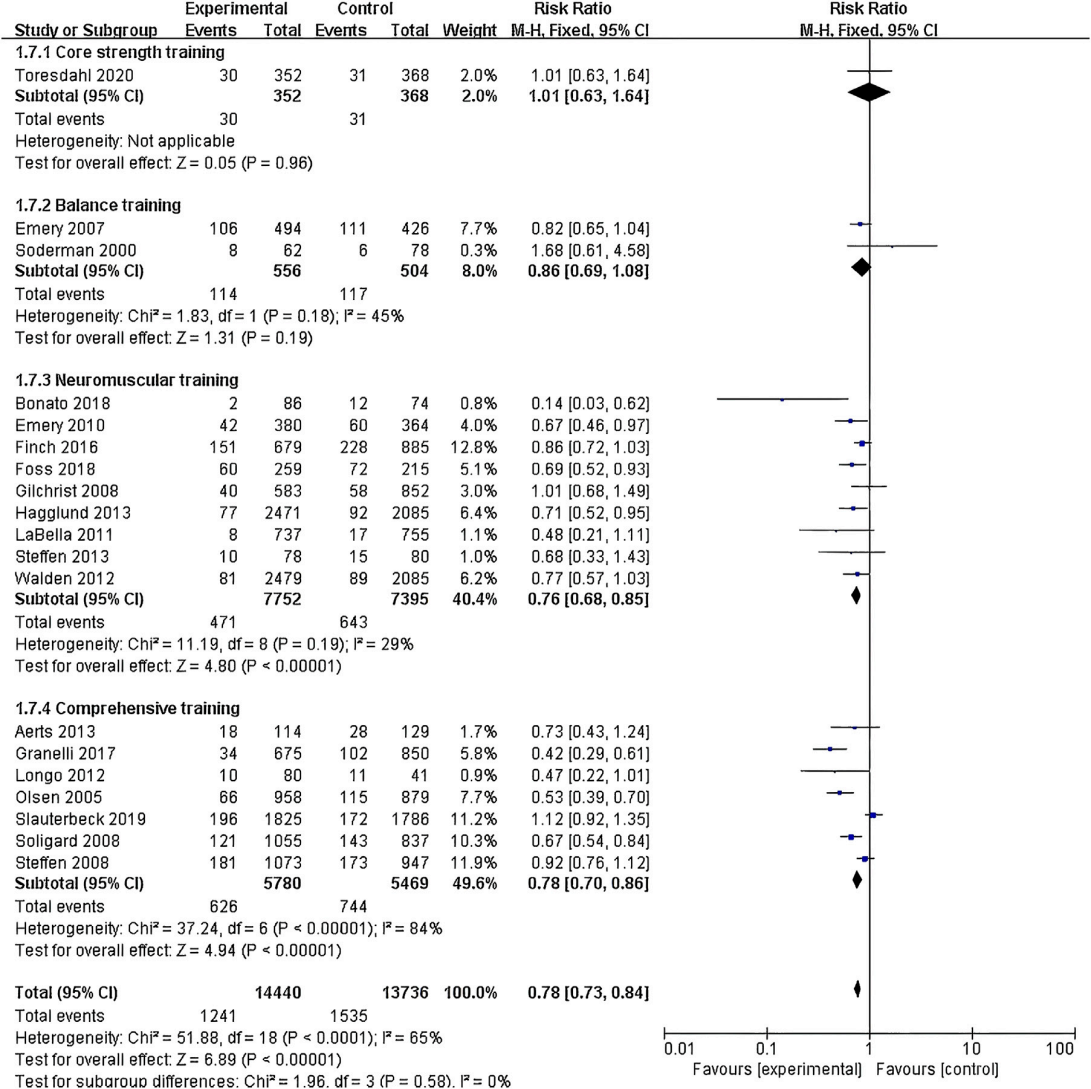
Forest plot of risk ratio by exercise intervention programs subgroup analysis.

**FIGURE 6:**
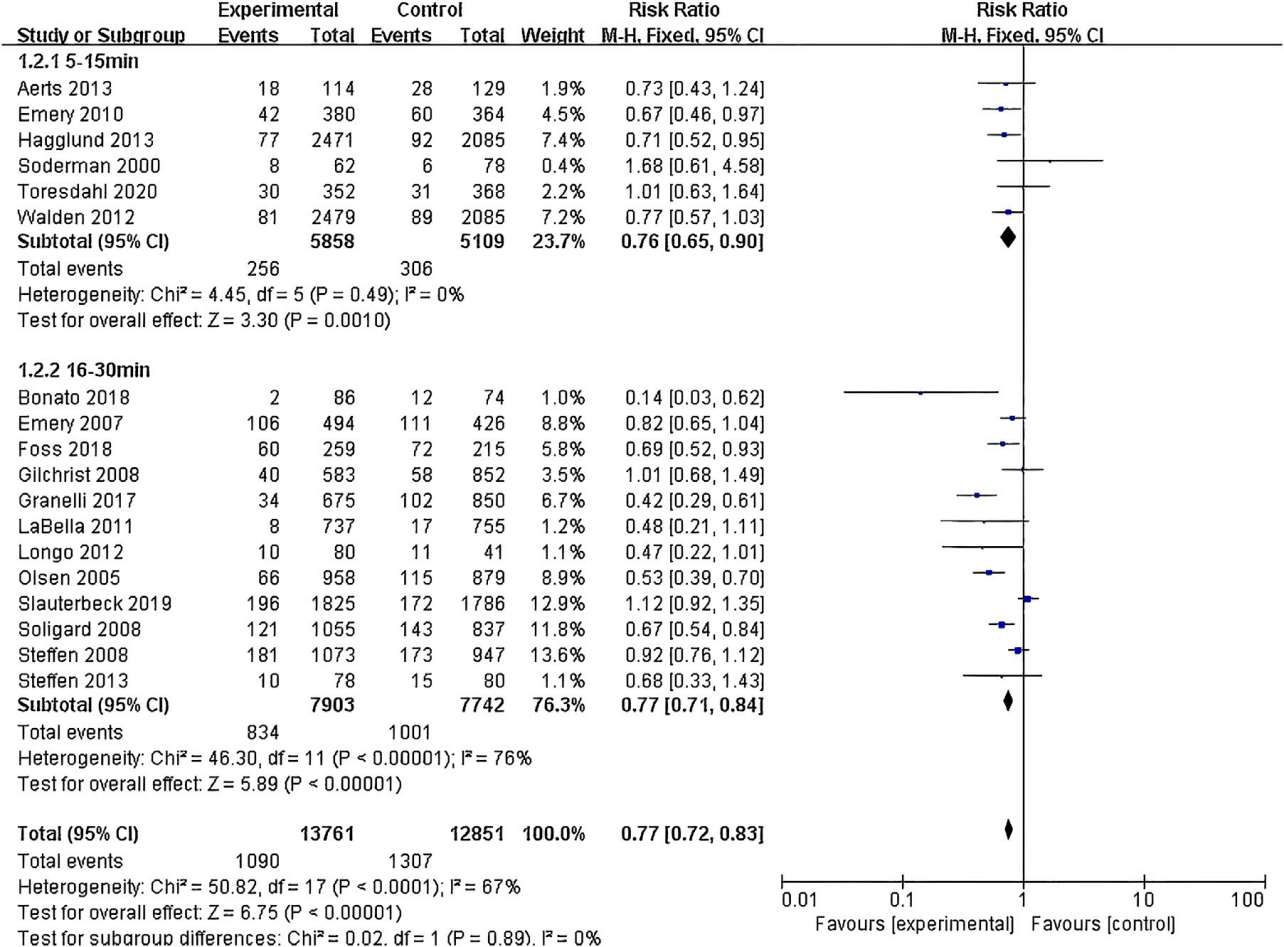
Forest plot of risk ratio by exercise duration subgroup analysis.

**FIGURE 7:**
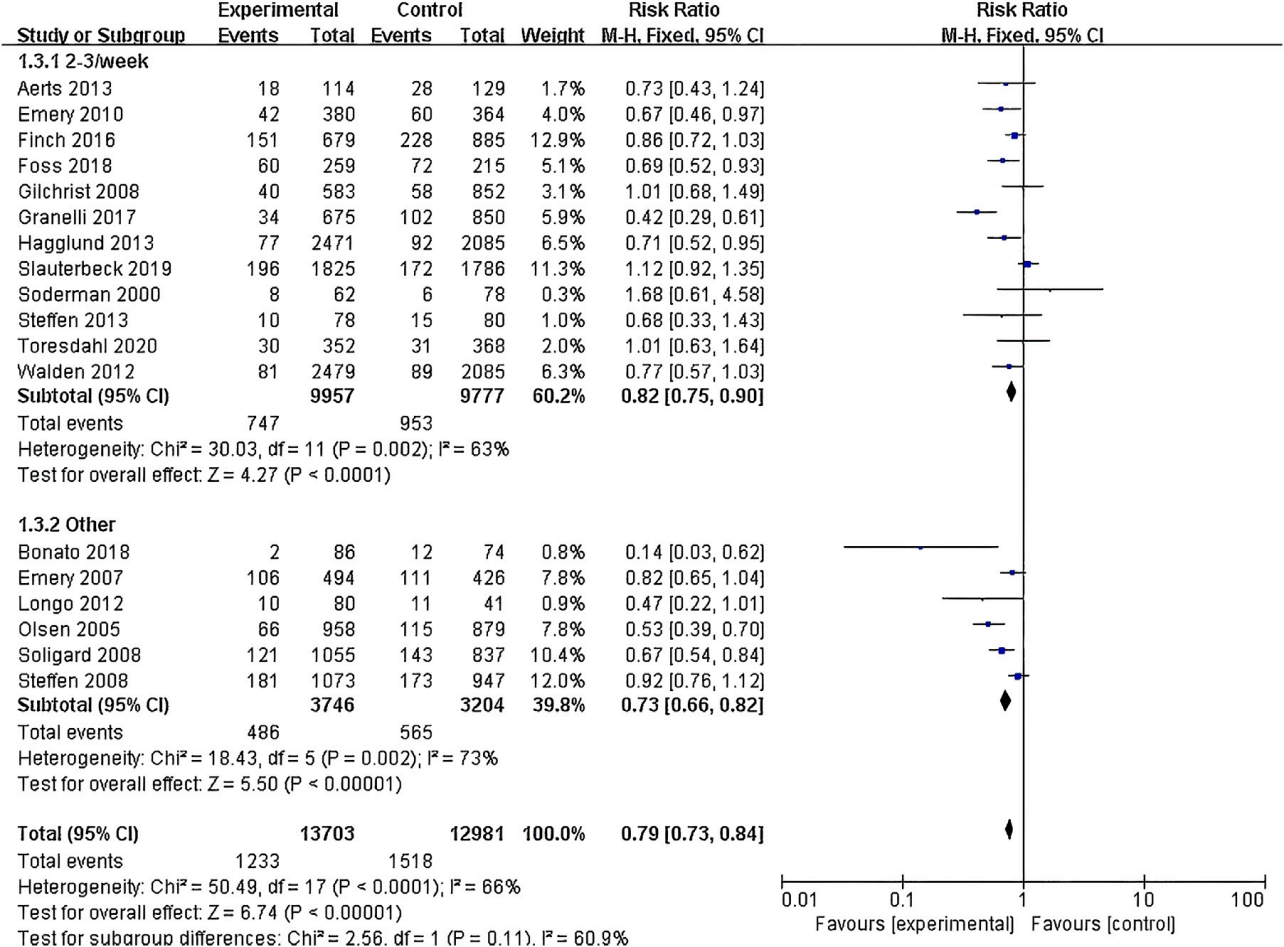
Forest plot of risk ratio by exercise frequency subgroup analysis.

**FIGURE 8:**
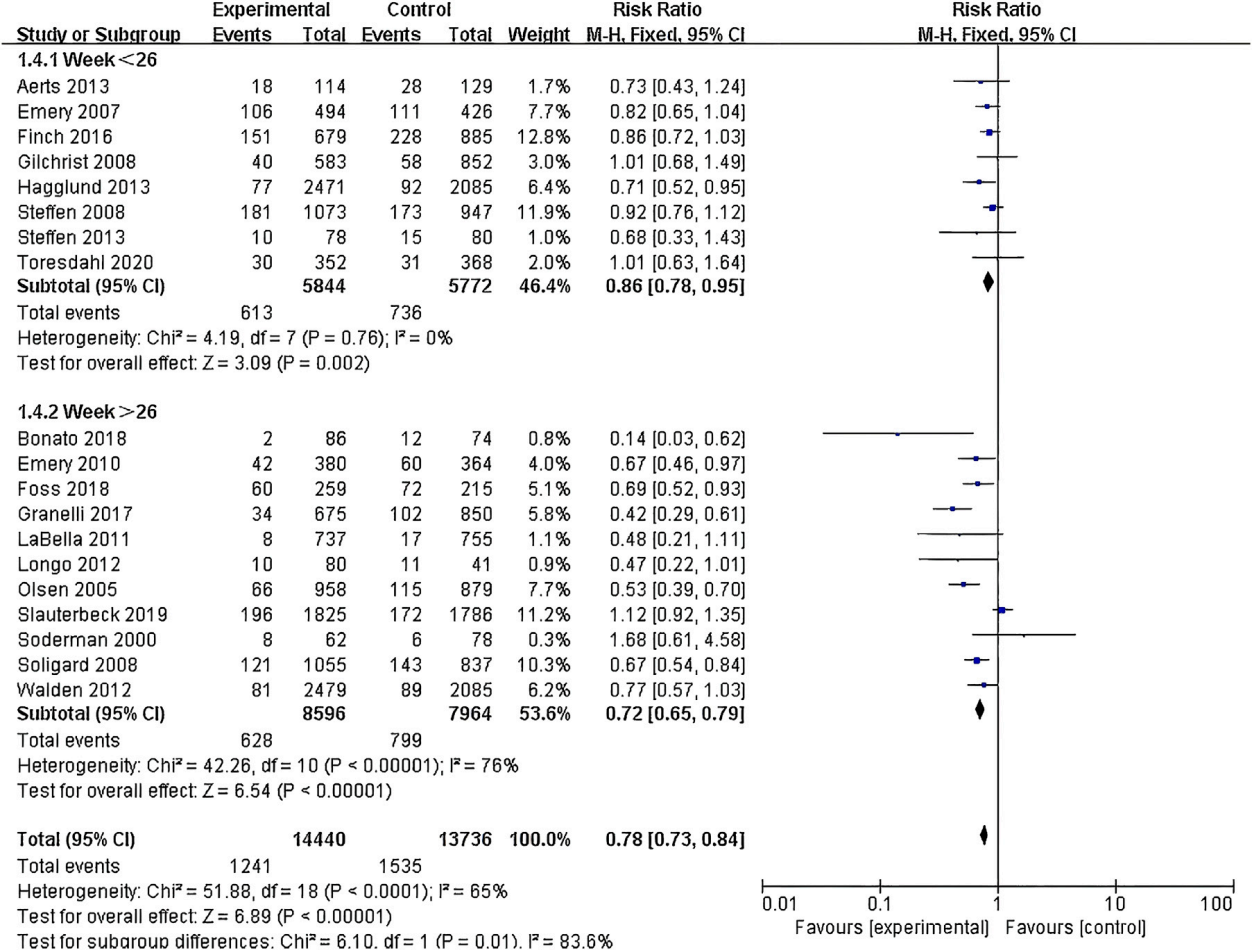
Forest plot of risk ratio by exercise period subgroup analysis.

**FIGURE 9:**
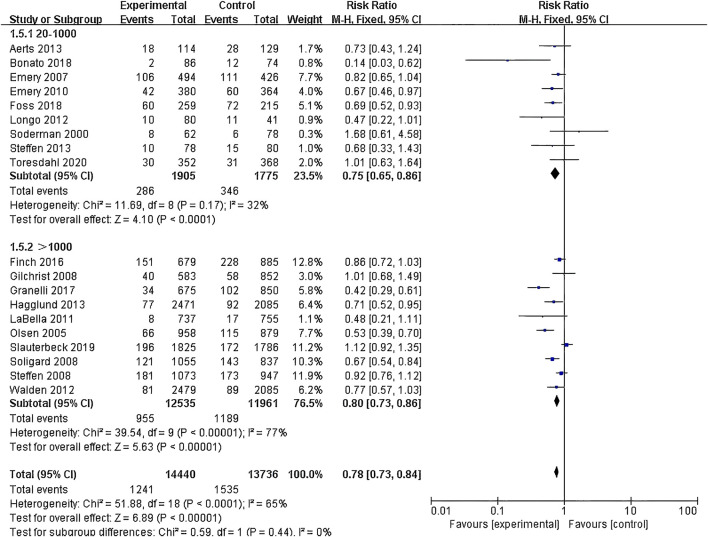
Forest plot of risk ratio by sample size subgroup analysis.

**FIGURE 10:**
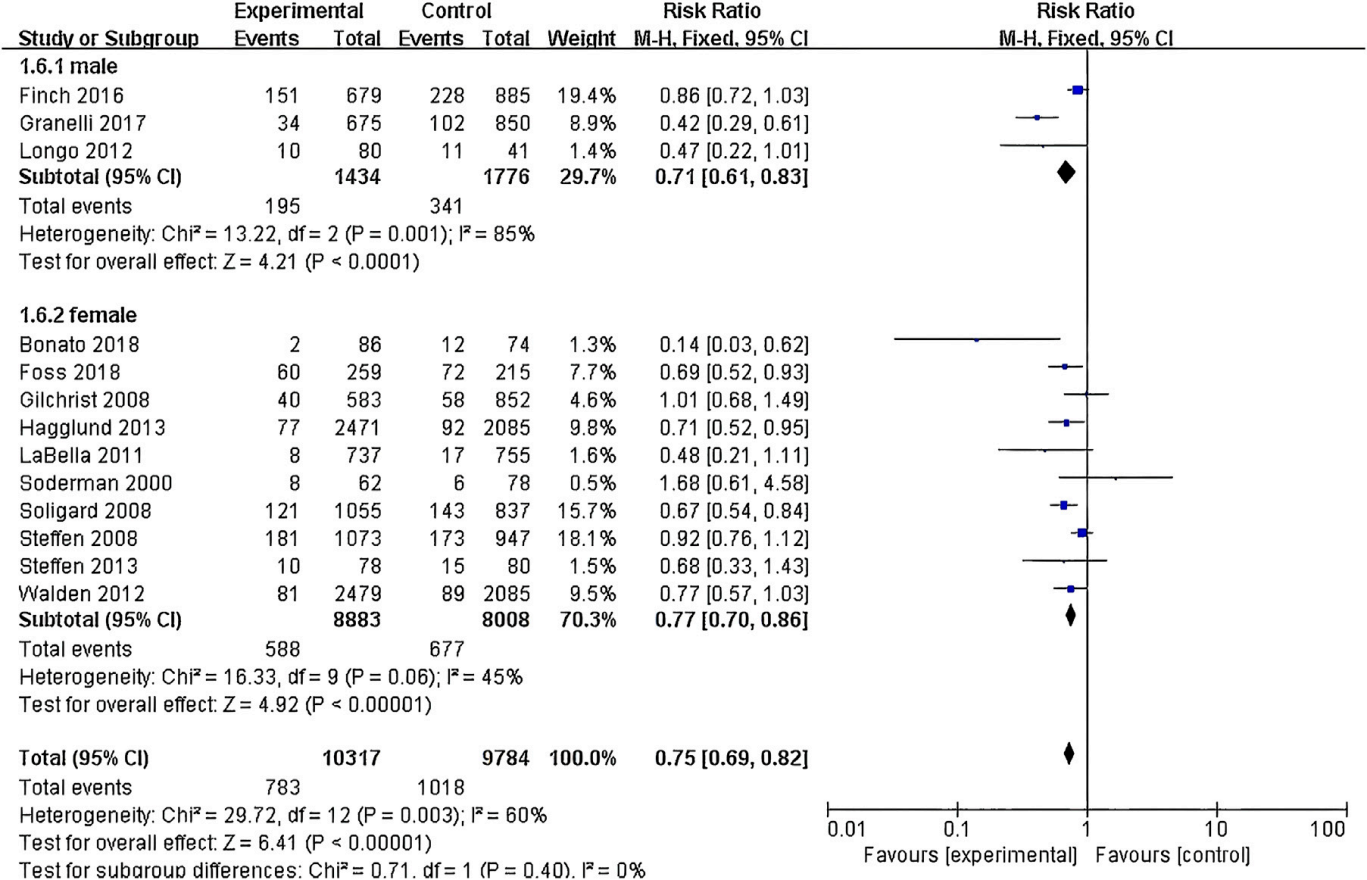
Forest plot of risk ratio by gender subgroup analysis.

(1) Exercise Intervention Programs: Subgroup analysis including 28,176 participants from the 19 RCT showed low heterogeneity within the neuromuscular training group (I^2^ = 29%) and high heterogeneity within the comprehensive training group (I^2^ = 84%). Both groups demonstrated statistically significant reductions in risk, with the neuromuscular training group showing a more pronounced effect size: RR = 0.76 (95% CI: 0.68–0.85; Z = 4.80; P < 0.00001).The core strength training group (P = 0.96) and balance training group (P = 0.19) had insufficient sample sizes, thus no statistical significance was observed (Figure 5; Table 3).
(2) Exercise Duration: Subgroup analysis involving 26,612 participants from 18 RCT was conducted. The results showed low heterogeneity within the 5–15 min group (I^2^ = 0%) and high heterogeneity within the 16–30 min group (I^2^ = 76%). Both the 5–15 min (P = 0.001) and 16–30 min (P < 0.001) groups showed statistically significant effect sizes. The 5–15 min group had a more pronounced effect size with RR = 0.76 (95% CI: 0.65–0.90; Z = 3.30; P = 0.001) and lower heterogeneity within the group (Figure 6).
(3) Exercise Frequency: In a subgroup analysis encompassing 26,684 participants from 18 RCT, there was observed moderate heterogeneity between the 2–3 times per week group and the other week group (I^2^ = 60.9%), with both groups showing moderate heterogeneity internally. Both the 2–3 times per week group and varying weekly frequencies group had statistically significant effect sizes (P < 0.001). Notably, the group with varied weekly frequencies exhibited a more substantial effect size, with RR = 0.73 (95% CI: 0.66–0.82; Z = 5.50; P < 0.00001), indicating a more pronounced reduction in risk (Figure 7).
(4) Exercise Period: Subgroup analysis including 28,176 participants from 19 RCT was conducted. The analysis identified high heterogeneity when comparing the group with exercise periods of 26 weeks or less to the group with periods exceeding 26 weeks, as indicated by an I^2^ of 83.6%. Despite the high heterogeneity, both duration groups exhibited statistically significant effect sizes (P < 0.001). The group with exercise periods longer than 26 weeks demonstrated a more substantial effect, with RR = 0.72 (95% CI: 0.65–0.79; Z = 6.54; P < 0.00001), suggesting a more pronounced reduction in risk associated with longer exercise periods (Figure 8).
(5) Sample Size: Subgroup analysis including 28,176 participants from 19 RCT articles showed low heterogeneity within the group of studies with sample sizes ranging from 20 to 1,000 participants (I^2^ = 32%). Conversely, high heterogeneity was present in the group with sample sizes above 1,000 participants (I^2^ = 77%). Despite the differing levels of heterogeneity, statistically significant effect sizes were found in both groups (P < 0.001). The group with 20–1,000 participants exhibited a more pronounced effect size, with RR = 0.75 (95% CI: 0.65–0.86; Z = 4.10; P < 0.0001), indicating a substantial reduction in risk within this range of sample sizes (Figure 9).
(6) Gender: Subgroup analysis involving 20,101 participants from 13 RCT indicated moderate heterogeneity within the female group (I^2^ = 45%) and high heterogeneity within the male group (I^2^ = 85%). Both groups showed statistically significant effect sizes (P < 0.001). The male group showed a more pronounced effect size, with RR = 0.71 (95% CI: 0.61–0.83; Z = 4.21; P < 0.00001) (Figure 10).

**TABLE 3:** Summary of subgroup analysis results.

Subgroup analysis	Heterogeneity			Grouping	Rate Ratio (95% CI)	Two-tailed test		Number of publications	Sample size
	I^2^ (%)		P-value			Z-value	P-value		
Intervention measures	—	—	—	Core strength training	1.01 [0.63, 1.64]	0.05	0.96	1	720
		45%	0.18	Balance training	0.86 [0.69, 1.08]	1.31	0.19	2	1,060
		29%	0.19	Neuromuscular training	0.76 [0.68, 0.85]	4.80	<0.00001	9	15,147
		84%	<0.00001	Comprehensive training	0.78 [0.70, 0.86]	4.94	<0.00001	7	11,249
Exercise duration	—	—	0.49	5–15 min	0.76 [0.65, 0.90]	3.30	0.0010	6	10,967
		76%	<0.00001	16–30 min	0.77 [0.71, 0.84]	5.89	<0.00001	12	15,645
Exercise frequency	60.9%	63%	0.002	2–3/week	0.82 [0.75, 0.90]	4.27	<0.0001	12	19,734
		73%	0.002	Other	0.73 [0.66, 0.82]	5.50	<0.00001	6	6,950
Exercise period	83.6%	—	0.76	Week ≤ 26	0.86 [0.78, 0.95]	3.09	0.002	8	11,616
		76%	<0.00001	Week > 26	0.72 [0.65, 0.79]	6.54	<0.00001	11	16,560
Sample size	—	32%	0.17	20–1,000	0.75 [0.65, 0.86]	4.10	<0.0001	9	3,680
		77%	<0.00001	>1,000	0.80 [0.73, 0.86]	5.63	<0.00001	10	24,496
Gender	—	85%	0.001	Male	0.71 [0.61, 0.83]	4.21	<0.0001	3	3,210
		45%	0.06	Female	0.77 [0.70, 0.86]	4.92	<0.00001	10	16,891

CI, Confidence Interval.

### Publication bias assessment

**FIGURE 11:**
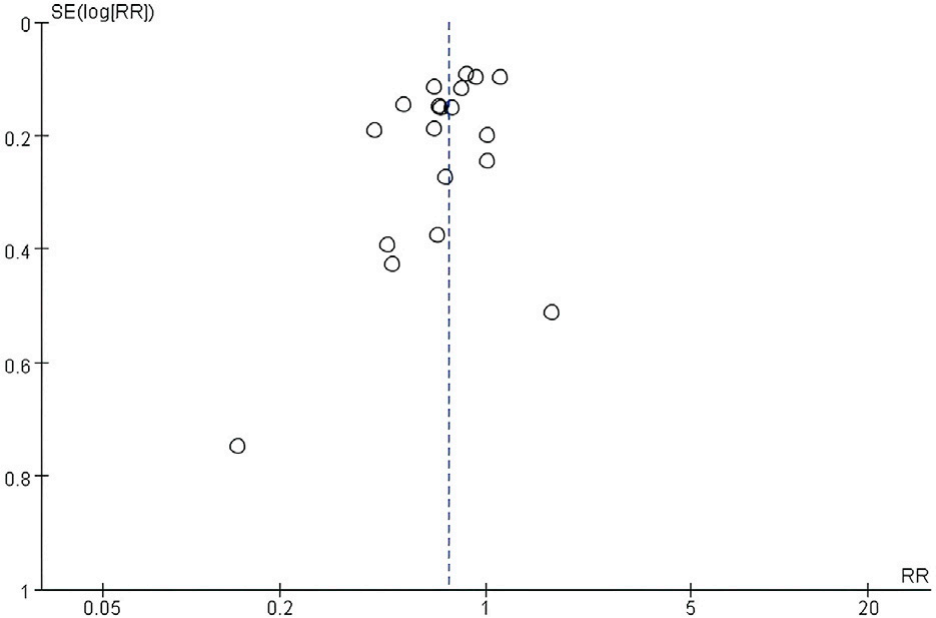
Funnel plot.

The publication bias assessment was conducted using a funnel plot to evaluate the effect of publication bias on the meta-analysis of the preventive effect of exercise intervention on lower limb injuries (Figure 11). The plot revealed a primarily symmetrical distribution of data points around the central axis, with most points clustered above the line. There were only two articles were dispersed below the line, indicating potential bias compared to other studies. The symmetry observed in the funnel plot generally indicates an absence of significant publication bias within the study sample, lending credence to the reliability of meta-analysis outcomes.

## Discussion

In this study, we conducted a meta-analysis of 19 RCTs to assess the effectiveness of exercise interventions in preventing lower limb knee injuries. The quantitative synthesis indicated that exercise interventions were effective in preventing lower limb knee injuries, with a pooled effect size (RR) of 0.75 (95% CI: 0.65–0.85; P < 0.001). Sensitivity analysis and subgroup analysis were performed on the included 19 studies to explore heterogeneity and identify the optimal intervention content, exercise duration, frequency, and period. The subgroup analysis of exercise intervention content revealed that all four intervention content types showed significant preventive effects on injuries. Previous studies have shown that core strength training can prevent ACL injuries by increasing the co-activation ratio of the hamstring and quadriceps muscles and reducing knee valgus and hip adduction angles (Jeong et al., 2021). Neuromuscular training, on the other hand, reduces the risk of sports-related injuries not only by increasing muscle strength and power but also by improving balance, speed, and agility (Zemková and Hamar, 2018). The intervention in the included neuromuscular training studies emphasized strength, agility, and balance, with a particular focus on plyometric exercises within the intervention, which were shown to significantly enhance voluntary motor control, awareness, and neuromuscular coordination. These characteristics distinguish neuromuscular training from isolated core strength training and balance training. Due to the limited sample size of core strength and balance interventions, no significant differences were observed. However, neuromuscular training intervention showed the best effect size (RR = 0.76; P < 0.001). Studies on different sports have indicated that exercise can enhance lower limb neuromuscular control, balance, muscle strength, and core strength, leading to the prevention of knee injuries. The occurrence of lower limb injuries is often associated with weak lower limb neuromuscular control, decreased balance, and insufficient core strength. Existing literature suggests that exercise-based prevention of lower limb knee injuries may be related to the improvement of these three factors (Akerlund et al., 2020; Andrew et al., 2013; Bulow et al., 2021; Jeong et al., 2021; Liu et al., 2016). Human movement not only involves mechanical structures but also requires a coordinated interplay between balance, neural control, and core strength. While it is important to examine the individual factors influencing knee injuries, it is also crucial to focus on their interrelationships. Core strength can improve the dynamic balance and coordination of the lower and upper limbs, thus reducing the risk of injury by maintaining the correct knee joint alignment (Affandi et al., 2019).

Enhanced balance, proprioception, and neuromuscular control may be associated with the prevention of lower limb knee injuries. A study involving 14 ACL-injured athletes examined the lower limb forward reach and three-dimensional force plate vertical jump tests after 3 and 6 months of rehabilitation treatment. The results showed that ACL injuries decreased the forward reach distance, peak jump and landing forces, and increased bilateral asymmetry, indicating that balance and strength were factors contributing to abnormal movement patterns (Dai et al., 2021). Muscle strength and balance are also correlated (Song et al., 2021). Hu conducted further research and found that lower limb strength is associated with dynamic postural stability. Improving dynamic postural stability through lower limb muscle strength training may reduce the risk of ACL injuries in the knee joint (Hu C. et al., 2023). The decline in neuromuscular control is also accompanied by changes in the structure of the lower limb knee joint, as reflected in the dynamic adaptation of the knee joint towards valgus alignment, which poses a significant risk for knee injuries (D’Onofrio et al., 2023). Following training interventions focused on balance and proprioception, rugby players experienced a 50% reduction in the occurrence of lower limb knee injuries (Attwood et al., 2018). A randomized controlled trial confirmed that specialized balance training in soccer could lower the incidence of hamstring and tendon injuries (Correia et al., 2020). This effect may be achieved by enhancing sensory input or accelerating neural conduction through balance and proprioception training, thereby improving the function and coordination of muscle-tendon units to better adapt to the upcoming exercise intensity. Furthermore, attention should be paid to muscle activation balance, as muscle imbalances are often reflected in significant differences in electromyography values. Earl’s study, which conducted electromyographic testing of six lower limb muscles during Y-balance exercises, demonstrated different activation patterns for muscles in different directions. Inner and inner posterior exercises can strengthen the quadriceps and help improve knee joint stability and ACL injury rehabilitation (Earl and Hertel, 2001). In addition to improving lower limb muscle balance, exercise can also increase hamstring muscle strength, optimize knee joint angles, and enhance posterior chain muscle strength, thereby improving the stability of the lower limb knee joint (Krommes et al., 2021).

The core strength is crucial throughout the entire process of body movement, transmitting upper and lower limb forces and regulating proper positioning of the center of gravity during exercise. Sasaki found that 8-week of continuous core muscle training can prevent lower limb injuries. This type of training can enhance athletes’ stability and control, reduce knee joint valgus torque, trunk flexion, and lateral inclination angles, and improve core strength, which is beneficial for preventing lower limb and exercise-related injuries, thus reducing biomechanical loads on the lower limbs and trunk (Sasaki et al., 2019). Guo found that dynamic core flexion strength can enhance countermovement jump height through arm swing, thereby improving athletic performance (Guo et al., 2020). Meanwhile, in terms of sports injuries, core stability plays a crucial role in preventing knee injuries during drop-jump landings (Guo et al., 2021). Training core strength and activating abdominal muscles effectively prevent lower limb injuries, especially non-contact ACL injuries. These methods improve neuromuscular control, reduce hip adduction angle and trunk lateral displacement, and reduce the risk of ACL injuries (Linde et al., 2018). Core strength training also enhances the trunk’s ability to resist perturbations, thereby reducing ACL load variables (Song et al., 2023). Saki concluded through an 8-week training that core strength training can improve kinematic characteristics, reduce high-risk landing mechanics, and prevent primary ACL injuries. It is recommended to perform core stability training after completing the postoperative rehabilitation plan to reduce the risk of re-injury (Saki et al., 2023). Therefore, by designing the content of core strength training during exercise, it is possible to improve the biomechanical structure of the lower limbs, enhance control over the lower limbs, and subsequently enhance control over the knee joint.

This study conducted subgroup analysis on exercise duration and found that both short durations of 5–15 min and moderate durations of 16–30 min can achieve the effect of preventing lower limb injuries. This is more feasible for various team sports and can be incorporated as warm-up exercises before different specialized training arrangements during the preseason or in-season. The study found that a short duration of 5–15 min achieved a better effect size (RR = 0.76; P = 0.001), providing the best preventive effect on lower limb sports injuries. This is consistent with the meta-analysis by Steib et al. (2017) on dose-response of neuromuscular training in preventing injuries in adolescent athletes, where a short duration of 10–15 min showed a better effect size than a moderate duration, consistent with the results of this study. On the one hand, this may be due to neuromuscular factors, and on the other hand, additional exercise interventions may increase the risk of knee joint injuries, as confirmed by the study by Savage et al. (2018), which showed that longer exercise durations lead to fatigue and increased knee joint flexion-extension torque, thereby increasing the risk of injuries.

Subgroup analysis of exercise frequency showed that interventions conducted in alternate weeks (4–5 times/week) had the greatest effect size in preventing lower limb knee joint injuries (RR = 0.73; P < 0.001). This may be because the exercise intervention serves as a warm-up before formal training, and a higher frequency of short-duration exercise interventions is needed to achieve the desired preventive effect on knee injuries. This is consistent with the study by Emery et al. (2022), which introduced neuromuscular training warm-up programs for basketball players and reduced their knee injury rate by 36%. A frequency of 4–5 times/week in exercise intervention can enhance neuromuscular control and increase lower limb muscle control, leading to injury prevention effects. In this study, the subgroup of exercise frequency at 4–5 times/week had high heterogeneity within the group, and only 6 studies were included. Further research on exercise frequency is needed in the future.

This study examines the impact of exercise duration on the relationship between exercise interventions and the prevention of lower limb knee injuries. Subgroup analysis based on exercise duration reveals that exercise interventions lasting more than 26 weeks yield a superior effect size (R = 0.72; P < 0.001). Consistent with previous research by Hu S. et al. (2023), this study demonstrates that exercise plans with a duration of 21–30 weeks in interventions targeting the hamstring muscles are more effective in injury prevention. The current results indicate that exercise interventions exceeding 26 weeks produce the optimal effects in preventing lower limb injuries. This outcome may be attributed to the optimization of neuromuscular control, balance, lower limb biomechanics, and core strength achieved through the 4–6-month lower limb injury prevention program.

Furthermore, subgroup analysis based on sample size indicates that the optimal effect size (RR = 0.75; P < 0.001) is achieved with sample sizes ranging from 20 to 1,000 subjects. This finding suggests that sample sizes within this range are more conducive to implementing intervention plans, as they result in relatively smaller data and measurement errors and lower within-group heterogeneity. Consequently, the results are more reliable, particularly in the context of group sports projects.

Female athletes are more predisposed to a higher risk of ACL injuries due to improper movement patterns (Ogasawara et al., 2024). Gender differences not only manifest in sports injuries but also impact the relationship between exercise interventions and lower limb knee injuries. Studies have demonstrated that exercise interventions effectively reduce non-contact anterior cruciate ligament injuries in female athletes (Mattu et al., 2022). However, subgroup analysis focusing on gender reveals that exercise interventions have a better effect size in preventing lower limb knee injuries in males (RR = 0.71; P < 0.001). This finding may be attributed to the limited number of male-specific studies (3 articles) compared to female-specific studies (10 articles) in this subgroup analysis, along with the higher heterogeneity within the male group. Future research should strive to include more high-quality literature to investigate the effects of exercise interventions on different genders.

## Limitations and future perspectives

Limited research on high-level adult athletes: Currently, studies on exercise interventions primarily focus on adolescent athletes in middle and high school (12–20 years old), while there is a lack of research on adult athletes involved in elite competitions. Considering the higher incidence of knee joint injuries in high-level competitive sports, investigating the effects of exercise interventions on high-level athletes would be of significant importance. Therefore, future studies should aim to include high-level adult athletes in their research.

Lack of standardized exercise protocols: There is currently no consensus on the specific design of exercise programs for lower limb injury prevention. Different sports may have varying training content and sequencing, making it difficult to determine which factors contribute most significantly to preventing knee injuries. The diversity in intervention content also complicates the interpretation of physiological mechanisms. Future research could incorporate neurophysiological and proprioceptive aspects to further investigate the underlying mechanisms. Additionally, quantitative analysis of exercise intensity and load, as well as the resulting metabolic stress on the body, should be conducted to better understand the effectiveness of lower limb injury prevention.

Biomechanical variations among different sports: The emphasis of exercise interventions may differ across various sports, which could introduce bias in the interpretation of biomechanical structures. Future studies should explore the characteristics of intervention content specific to each sport to gain a better understanding of their effects on injury prevention.

Insufficient inclusion of high-quality literature: The limited number of high-quality studies included in the analysis may contribute to heterogeneity among studies. Therefore, future research should prioritize high-quality studies to enhance the reliability of findings regarding the effectiveness of exercise interventions.

## Conclusion

We advocate for the integration of neuromuscular training into programs designed to prevent lower limb knee injuries. This approach is identified as a foundational strategy in ensuring knee health. The efficacy of such programs is maximized when they include exercises lasting 5–15 min, conducted 4–5 times per week, over a period extending beyond 26 weeks. This regimen not only offers a practical solution but also stands as an effective method to significantly lower the incidence of knee injuries. By adopting this evidence-based protocol, practitioners can better safeguard the knee health of athletes and individuals engaged in physical activities, ensuring long-term musculoskeletal wellbeing.

## Data Availability

The original contributions presented in the study are included in the article/Supplementary Material, further inquiries can be directed to the corresponding author.
