# Supplementary material for: The effects of training intervention on the prevention of knee joint injuries: a systematic review and meta-analysis
Source: Front Physiol. 2025 Feb 24;16:1455055. doi: 10.3389/fphys.2025.1455055 (PMC11891182; doi:10.3389/fphys.2025.1455055)
Supplement: Supplementary file 2 [file DataSheet1.docx]

**The PICOS Search Strategy**

| Population | Athletes with sports experience (male/female, age 12-20 years old) |
| --- | --- |
| Intervention | Exercise training |
| Comparison | Intervention group/control group |
| Outcome | Lower extremity sports injuries (anterior and posterior cruciate ligament injuries, medial and lateral collateral ligament injuries, meniscus injuries, cartilage injuries, patellar injuries) |
| Study design | Randomized controlled trials (RCTs) |

**Search terms**

**(1)Pubmed (Until 2024/04/12)**

Search: ((prevent*[Title/Abstract]) AND (((((((((((((((((((((((((((Exercises[Title/Abstract]) OR (Physical Activity[Title/Abstract])) OR (Activities, Physical[Title/Abstract])) OR (Activity, Physical[Title/Abstract])) OR (Physical Activities[Title/Abstract])) OR (Exercise, Physical[Title/Abstract])) OR (Exercises, Physical[Title/Abstract])) OR (Physical Exercise[Title/Abstract])) OR (Physical Exercises[Title/Abstract])) OR (Acute Exercise[Title/Abstract])) OR (Acute Exercises[Title/Abstract])) OR (Exercise, Acute[Title/Abstract])) OR (Exercises, Acute[Title/Abstract])) OR (Exercise, Isometric[Title/Abstract])) OR (Exercises, Isometric[Title/Abstract])) OR (Isometric Exercises[Title/Abstract])) OR (Isometric Exercise[Title/Abstract])) OR (Exercise, Aerobic[Title/Abstract])) OR (Aerobic Exercise[Title/Abstract])) OR (Aerobic Exercises[Title/Abstract])) OR (Exercises, Aerobic[Title/Abstract])) OR (Exercise Training[Title/Abstract])) OR (Exercise Trainings[Title/Abstract])) OR (Training, Exercise[Title/Abstract])) OR (Trainings, Exercise[Title/Abstract])) OR (Sports[Title/Abstract])) OR (Sport[Title/Abstract]))) AND (((((((((((((((((((((((((((((((Lower extremity injuries[Title/Abstract]) OR (Knee Injuries[Title/Abstract])) OR (Injury, Knee[Title/Abstract])) OR (Meniscus[Title/Abstract])) OR (Menisci[Title/Abstract])) OR (Medial Collateral Ligament, Knee[Title/Abstract])) OR (Medial Ligament of Knee[Title/Abstract])) OR (Knee Medial Ligament[Title/Abstract])) OR (Knee Medial Ligaments[Title/Abstract])) OR (Lateral collateral ligament[Title/Abstract])) OR (Anterior Cruciate Ligament[Title/Abstract])) OR (Cruciate Ligament, Anterior[Title/Abstract])) OR (Anterior Cruciate Ligaments[Title/Abstract])) OR (Cruciate Ligaments, Anterior[Title/Abstract])) OR (Ligament, Anterior Cruciate[Title/Abstract])) OR (Ligaments, Anterior Cruciate[Title/Abstract])) OR (Anterior Cranial Cruciate Ligament[Title/Abstract])) OR (Cranial Cruciate Ligament[Title/Abstract])) OR (Cranial Cruciate Ligaments[Title/Abstract])) OR (Cruciate Ligament, Cranial[Title/Abstract])) OR (Cruciate Ligaments, Cranial[Title/Abstract])) OR (Ligament, Cranial Cruciate[Title/Abstract])) OR (Ligaments, Cranial Cruciate[Title/Abstract])) OR (Cartilage[Title/Abstract])) OR (Cartilages[Title/Abstract])) OR (Patella[Title/Abstract])) OR (Patellas[Title/Abstract])) OR (Kneecap[Title/Abstract])) OR (Kneecaps[Title/Abstract])) OR (Knee Cap[Title/Abstract])) OR (Knee Caps[Title/Abstract])) Filters: from 2000 - 2024

*After retrieving, replace with "reduces" and conduct another search

**(2)Web of Science (Until 2024/04/12)**

Prevent*

<https://www.webofscience.com/wos/alldb/summary/399f1d51-9817-4512-9dda-503007354439-815fb609/relevance/1>

Reduce

https://www.webofscience.com/wos/alldb/summary/d2625b67-8f55-461c-8266-0ba09d39ba4b-815fdde3/relevance/1

*After retrieving, replace with "reduces" and conduct another search

**(3)EBSCO (Until 2024/04/12)**

Search Alert: "( Lower extremity injuries or Knee Caps or Knee Cap or Kneecaps or Kneecap or Patellas or Patella or Cartilages or Cartilage or Ligaments, Cranial Cruciate or Ligament, Cranial Cruciate or Cruciate Ligaments, Cranial or Cruciate Ligament, Cranial or Cranial Cruciate Ligaments or Cranial Cruciate Ligament or Anterior Cranial Cruciate Ligament or Ligaments, Anterior Cruciate or Ligament, Anterior Cruciate or Cruciate Ligaments, Anterior or Anterior Cruciate Ligaments or Cruciate Ligament, Anterior or Anterior Cruciate Ligament or Lateral collateral ligament or Knee Medial Ligaments or Knee Medial Ligament or Medial Ligament of Knee or Medial Collateral Ligament, Knee or Menisci or Meniscus or Injury, Knee or Knee Injuries or Injuries, Knee ) AND ( Exercises or Sport or Sports or Trainings, Exercise or Training, Exercise or Exercise Trainings or Exercise Training or Exercises, Aerobic or Aerobic Exercises or Aerobic Exercise or Exercise, Aerobic or Isometric Exercise or Isometric Exercises or Exercises, Isometric or Exercise, Isometric or Exercises, Acute or Exercise, Acute or Acute Exercises or Acute Exercise or Physical Exercises or Physical Exercise or Exercises, Physical or Exercise, Physical or Physical Activities or Activity, Physical or Physical Activity or Activities, Physical or ) AND prevent* Full Text; Published Date: 20000101-20240412 AND Apply equivalent subjects on 2024-04-12 02:29 PM"

*After retrieving, replace with "reduces" and conduct another search

**(3)China National Knowledge Infrastructure (Until 2024/04/12)**

检索式：

( ( ( ( ( ( ( ( ( ( 主题%='半月板' or 题名%='半月板' ) OR ( 主题%='膝关节损伤' or 题名%='膝关节损伤' ) ) OR ( 主题%='内侧副韧带' or 题名%='内侧副韧带' ) ) OR ( 主题%='外侧副韧带' or 题名%='外侧副韧带' ) ) OR ( 主题%='前交叉韧带' or 题名%='前交叉韧带' ) ) OR ( 主题%='软骨损伤' or 题名%='软骨损伤' ) ) OR ( 主题%='髌骨劳损' or 题名%='髌骨劳损' ) ) AND ( ( ( 主题%='运动' or 题名%='运动' ) OR ( 主题%='锻炼' or 题名%='锻炼' ) ) OR ( 主题%='训练' or 题名%='训练' ) ) ) AND ( 主题%='预防' or 题名%='预防' ) *) AND ( ( ( 摘要=xls('RCT') ) OR ( 摘要='随机对照' ) ) OR ( 摘要='随机' ) ) )

*After retrieving, replace with "reduces" and conduct another search

**(4)Wanfang Database (Until 2024/04/12)**

检索表达式： (主题:(下肢损伤 or 半月板 or 膝内侧副韧带 or 膝外侧副韧带 or 前交叉韧带 or 软骨损伤 or 髌骨劳损) and 主题:(运动 or 锻炼 or 训练) and 主题:(预防*)) and Date:2000-2024

*After retrieving, replace with "reduces" and conduct another search
